# New insights into crosstalk between Nrf2 pathway and ferroptosis in lung disease

**DOI:** 10.1038/s41419-024-07224-1

**Published:** 2024-11-18

**Authors:** Yonghu Chen, Zhe Jiang, Xuezheng Li

**Affiliations:** College of Pharmacy, Yanbian University Hospital, Yanbian University, Yanji, 133002 P. R. China

**Keywords:** Cell death, Mechanisms of disease, Pharmacology

## Abstract

Ferroptosis is a distinctive process of cellular demise that is linked to amino acid metabolism, lipid oxidation, and iron oxidation. The ferroptosis cascade genes, which are closely associated with the onset of lung diseases, are among the regulatory targets of nuclear factor erythroid 2-related factor 2 (Nrf2). Although the regulation of ferroptosis is mostly mediated by Nrf2, the precise roles and underlying regulatory mechanisms of ferroptosis and Nrf2 in lung illness remain unclear. This review provides new insights from recent discoveries involving the modulation of Nrf2 and ferroptosis in a range of lung diseases. It also systematically describes regulatory mechanisms involving lipid peroxidation, intracellular antioxidant levels, ubiquitination of Nrf2, and expression of FSP1 and GPX4. Finally, it summarises active ingredients and drugs with potential for the treatment of lung diseases. With the overarching aim of expediting improvements in treatment, this review provides a reference for novel therapeutic mechanisms and offers suggestions for the development of new medications for a variety of lung disorders.

## Facts

- Nrf2 plays a pivotal role in the regulation of essential genes involved in ferroptosis and is intimately linked to the progression of lung diseases.
- In lung cancer, the hyperactivation of Nrf2 impedes the induction of ferroptosis in malignant cells, thereby conferring a survival advantage.
- The creation of pharmaceutical agents aimed at either stimulating or impeding the activity of Nrf2 proteins represents a promising strategy for modulating ferroptosis.
- The combined use of Nrf2 inhibitors enhances the sensitivity of lung cancer cells to ferroptosis.

## Open questions

- What are the potential limitations and challenges associated with targeting Nrf2 inhibition to induce ferroptosis as an effective treatment for lung cancer?
- Are there any unexplored potential targets for the crosstalk between Nrf2 and ferroptosis?
- What serves as the key hub connecting Nrf2 and ferroptosis?
- What is the clinical efficacy of drugs targeting Nrf2 and ferroptosis, for the treatment of lung diseases?

## Introduction

Lung diseases are a complex and diverse group of pathological states that severely affect the structure and function of the lungs and have become a major health threat worldwide. Pulmonary diseases include chronic obstructive pulmonary disease (COPD), interstitial lung diseases (ILDs), infectious lung diseases, and lung cancer, with the global incidence rates of COPD and lung cancer showing alarming increases year by year [1–3]. Currently, COPD is the third most common fatal disease worldwide, while lung cancer has one of the highest cancer-related mortality rates [4, 5]. Oxidative stress is thought to play a central role in the pathogenesis of these lung diseases. Oxidative stress directly damages lipids, proteins, and DNA in lung tissues, induces an inflammatory response, and also further accelerates the pathological process by inducing iron death in lung tissues [6]. Prolonged exposure to irritants such as tobacco smoke leads to a sustained increase in oxidative stress in the lungs of COPD patients, which in turn inhibits the function of the nuclear factor erythroid 2-related factor 2 (Nrf2) pathway and weakens the body’s antioxidant defences, leading to further damage to alveolar structure, increased airway remodelling, and inflammatory responses, thus driving the pathological progression of the disease [7]. Similarly, in lung fibrosis, inhibition of the Nrf2 pathway is closely associated with elevated levels of oxidative stress in fibrotic tissues, which in turn triggers the abnormal deposition of fibrous components such as collagen, leading to a post-ferroptotic state in lung tissue cells [8, 9]. Additionally, in lung cancer, hyperactivation of Nrf2 enables cancer cells to regulate intracellular iron metabolism more effectively, thereby inhibiting iron-dependent lipid peroxidation and evading ferroptosis [10]. This mechanism not only helps cancer cells survive a hostile environment, it also confers tolerance to treatments such as radiotherapy and chemotherapy, and is recognised as an important cause of drug resistance during lung cancer treatment. It is evident that dysregulation of the Nrf2 pathway and regulation of the inflammatory response and ferroptosis play key roles in the pathogenesis of several lung diseases, making Nrf2 a potential therapeutic target. Elucidating the regulatory mechanisms of Nrf2 and ferroptosis in lung diseases could profoundly deepen our understanding of the pathogenesis of these diseases and inspire new research directions for their treatment.

**Fig. 1:**
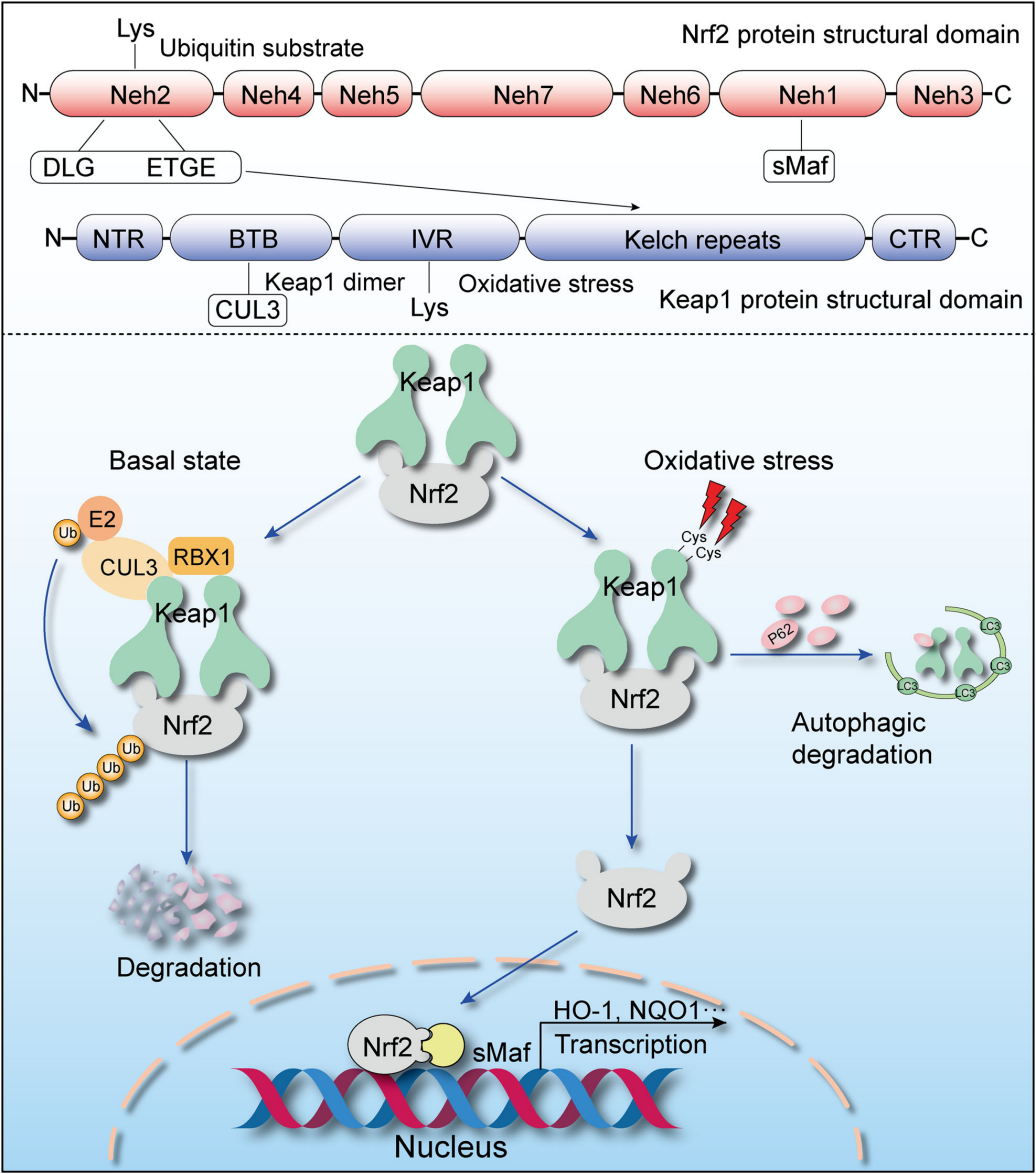
Keap1-mediated regulation of Nrf2 through its Neh structural domains under normal and oxidative stress conditions.

Since its discovery in 1994, Nrf2 has been widely studied and shown to be an essential controller of antioxidant genes. Additionally, we now know the precise molecular makeup and mode of regulation of its pathway partner, Kelch-like ECH-associated protein 1 (Keap1) [11, 12]. Among the seven conserved functional areas referred to as the Nrf2-ECH homology (Neh) domains of Nrf2, Neh1 primarily dimerises and attaches to small Maf (sMAF) proteins before going on to bind to DNA [13, 14]. Neh2 is the key to stable Keap1 binding, while two Neh2 motifs, hinge ETGE and latch DLG, interact with Keap1 in a hinge-and-latch mechanism [15]. Additionally, the Neh2 structural domain contains lysine residues, which are substrates for ubiquitination and are involved in the degradation of the Nrf2 protein [16]. Neh3, Neh4, and Neh5 are involved in transactivation, while Neh6 regulates self-stability [17]. Keap1, as a negative regulator of Nrf2, comprises three main structural and functional domains: BTB, IVR, and Kelch repeat [18]. The BTB domain binds CUL3 and is used to link to E3 ubiquitin ligase, the IVR domain regulates cysteine residue activity, and the Kelch repeat domain is mainly responsible for linking to Nrf2 (Fig. 1) [19, 20].

In the basal state, Nrf2 is mostly found in the cytoplasm of cells, bound to Keap1. Nrf2 relies on proteasomal degradation to maintain low intracellular levels, which is mediated by Keap1. Keap1, in association with CUL3 and Rbx1, forms a functional E3 ubiquitin ligase that ubiquitinates Nrf2, targeting it for degradation [21, 22]. Under oxidative stress conditions, active cysteine residues of Keap1 undergo direct modifications, changing the structure of the active E3 ubiquitin ligase and leading to a breakdown in ubiquitination [23, 24]. Nrf2 then accumulates in the cytoplasm and translocates to the nucleus [25], where the Nrf2-dependent self Neh1 attaches to sMAF transcription factors. Nrf2-sMAF heterodimers ultimately bind to antioxidant response elements (AREs), which control the expression of anti-inflammatory, cytoprotective, and antioxidant proteins.

**Fig. 2:**
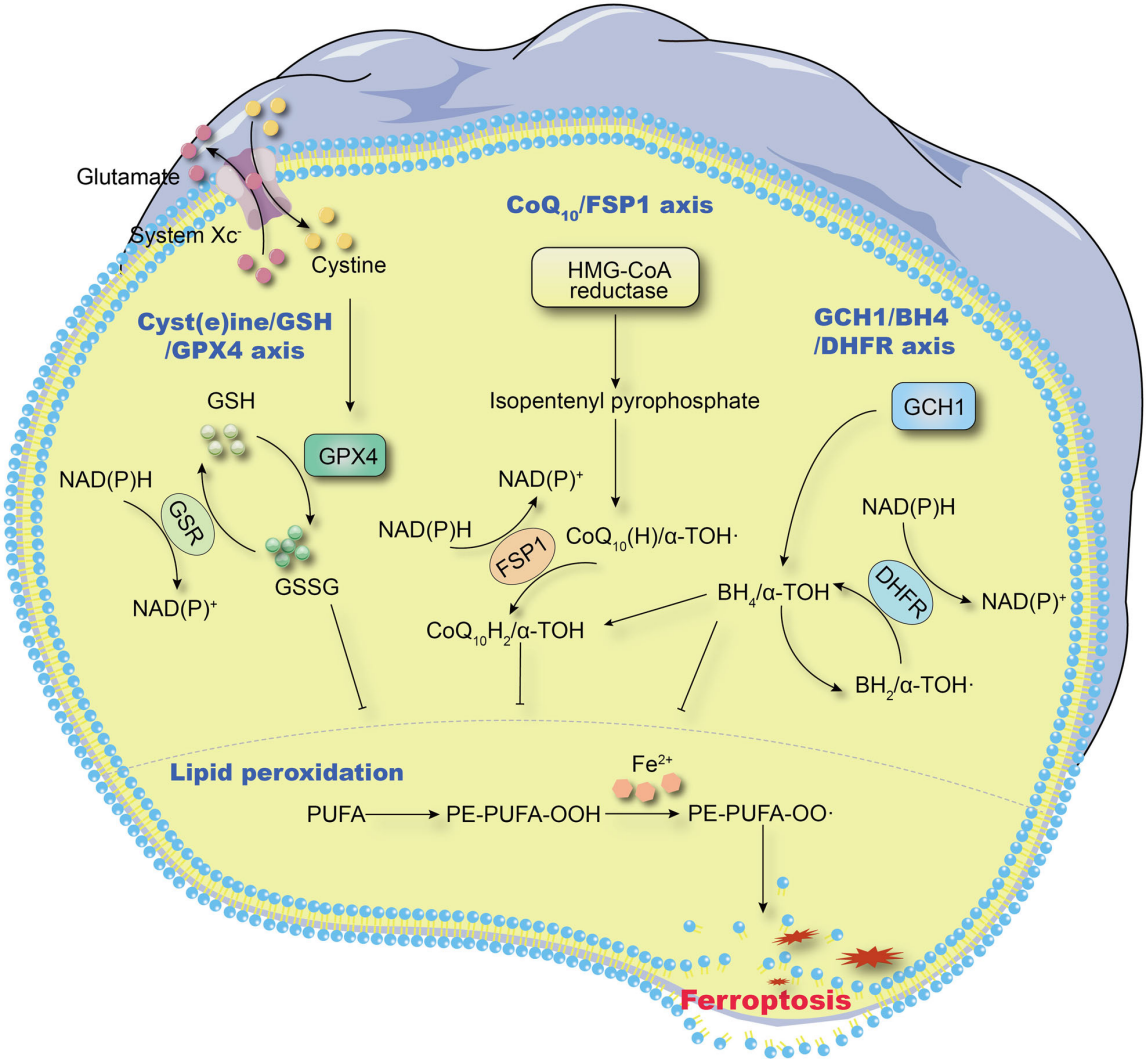
Activation and regulatory mechanisms of ferroptosis. DHFR, dihydrofolate reductase; GSSG, oxidised glutathione (GSH); GSR, GSH reductase; PE, phosphatidylethanolamine.

Since its identification in 2012, the mechanisms underlying ferroptosis have gradually been unravelled. Unlike other forms of regulated cell death, ferroptosis is an iron-dependent, non-apoptotic process [26, 27] characterised by increased reactive oxygen species (ROS) and lipid peroxidation, both of which are driven by the accumulation of intracellular iron [28–30]. Lipid peroxidation, damage to the plasma membrane, fewer mitochondria, diminished mitochondrial cristae, and increased membrane density are the primary characteristics of ferroptosis [31, 32]. Thorough mechanistic investigations have recently revealed that multiple metabolic pathways linked with the process of lipid peroxidation ultimately impact the regulation of ferroptosis (Fig. 2). Among these, the ferroptosis process known as the cyst(e)ine/glutathione (GSH)/glutathione peroxidase (GPX)4 axis is regulated by the classical GPX4. In this mechanism, the GSH-dependent cystine reduction pathway stimulates the production of GSH by reducing the cysteine that system Xc transports into the cell [33–35]. GPX4 requires GSH, a strong reducing agent, as a cofactor to effectively reduce phospholipid hydroperoxides (PLOOHs) to their equivalent alcohols (PLOHs) inside cells. Reducing PLOOH buildup shields cells from ferroptosis, delaying the quick and irreversible destruction to membrane components [36, 37]. Although the main factor in ferroptosis prevention is believed to be GPX4, other crucial players include the GPX4-independent ferroptosis suppressor protein 1 (FSP1) [38]. It was discovered that FSP1 (originally known as AIFM2) shields cells from GPX4-independent ferroptosis brought on by suppression or deletion of GPX4. FSP1 accomplishes this through its ubiquinone oxidoreductase activity, which produces panthenol by decreasing the incomplete oxidation product of ubiquinone (CoQ10), semihydroquinone [39]. This free radical is able to directly lower lipid free radicals and stop lipid autoxidation, or indirectly promote the regeneration of oxidised α-tocopherol free radicals, which are the most potent naturally occurring chain-breaking antioxidants in lipids [40]. Additionally, it has been revealed that GTP cyclohydrolase 1 (GCH1), through its metabolites tetrahydrobiopterin (BH4) and dihydrobiopterin (BH2), is resistant to ferroptosis [41]. BH4 acts both as a direct trap for small molecule free radicals and a cofactor for enzymes involved in CoQ10 synthesis, protecting phospholipids containing two polyunsaturated fatty acid (PUFA) tails from oxidative degradation during ferroptosis.

## Crosstalk between Nrf2 and ferroptosis

As an important stress factor that maintains intracellular oxidative homoeostasis, Nrf2 controls the expression of many antioxidant genes. Iron ions are essential for cell metabolism and survival, but excess free iron ions lead to increased intracellular oxidative stress, which promotes cell death, especially ferroptosis [42, 43]. Studies have shown that Nrf2 plays a crucial role in maintaining the levels and distribution of intracellular iron ions by regulating the gene expression of key factors involved in iron metabolism, including transferrin (Tf), ferritin, ferroportin, and FSP1 [44–46]. Activation of the Nrf2 pathway was found to diminish liver injury due to iron overload by regulating the expression of Tf and FTH and reducing the accumulation of cellular iron-free ions, thereby inhibiting oxidative stress and attenuating cell death [47]. Furthermore, Nrf2 activators reduce the extent of neuronal death caused by iron overload, most likely by regulating genes associated with iron metabolism and the synthesis of antioxidants that protect cells from oxidative stress and ferroptosis [48, 49]. One of these Nrf2 targets, haem oxygenase-1 (HO-1), has two purposes in ferroptosis: promotion of ferroptosis via the generation of Fe^2+^ from haem degradation, and deceleration of ferroptosis through the inhibition of oxidative stress. Thus, the dual functions of HO-1 are dependent on the balance between oxidative stress and ferroptosis [50–52]. Additionally, ferroptosis is considered to represent a new approach to anti-tumour therapy, and has been reported in lung cancer. However, deletion of *Keap1* activates high expression of Nrf2, contributing to tumour resistance to treatment [53, 54]. Along with decreasing oxidative stress in tumours, Nrf2 activation increases the expression of downstream genes *FSP1* and *GPX4*, which can shield cancerous cells from iron ptosis [55, 56]. Indeed, many lung cancer therapies are based on reducing Nrf2 expression to induce the sensitisation of lung cancer cells to ferroptosis. Elucidating how Nrf2 preferentially upregulates target genes to avert various forms of oxidative cell death could facilitate the design of new treatments and preventative strategies for iron-associated illnesses.

## Role of the Nrf2 pathway and ferroptosis in Acute Lung Injury (ALI)

### Novel mechanisms for regulating the Nrf2 pathway and ferroptosis in ALI

**Fig. 3:**
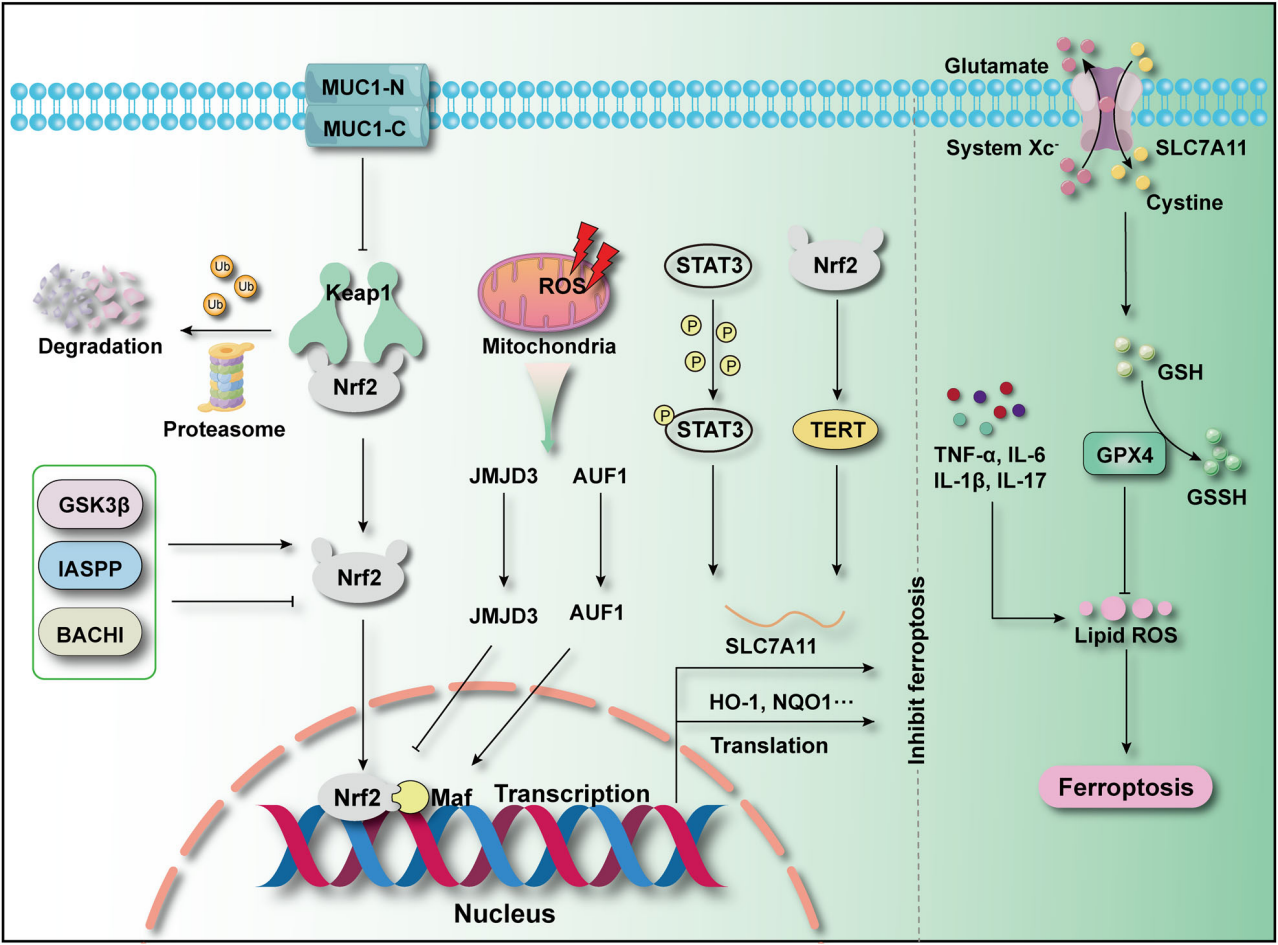
Mechanisms by which the Nrf2 signalling pathway is regulated to inhibit ferroptosis in ALI.

ALI is a common pathological state that is induced by a variety of factors, such as infection, trauma, and inhalation of harmful substances [57]. The pathological process of ALI involves extensive lung tissue damage and dysfunction, and is therefore more appropriately regarded as a clinical syndrome of acute lung injury rather than a stand-alone chronic lung disease [58]. In intensive care patients, ALI, especially acute respiratory distress syndrome (ARDS), is characterised by rapid deterioration, resulting in severe respiratory failure, morbidity, and death [59]. Preventive and specific therapeutic options for ALI are lacking, and constitute one of the major scientific problems in the field of respiratory critical care [60]. An in-depth study of ALI pathogenesis not only provided a scientific basis for new therapeutic strategies for a wide range of lung diseases, it also revealed the common pathological mechanisms of acute and chronic lung injury, including oxidative stress, inflammatory response, and ferroptosis [61]. Therefore, elucidation of the mechanisms of Nrf2 and ferroptosis in the regulation of ALI could be significant. Studies have shown that Nrf2-regulated ferroptosis plays important roles in the treatment and protection against ALI. In intestinal ischaemia-reperfusion (IIR-ALI)-induced ALI, knockdown of *Nrf2* exacerbated ALI injury, further reducing GPX4 expression and relative GSH content [62]. In contrast, ALI symptoms were lessened by increased expression of Nrf2. Furthermore, it was discovered that solute carrier family 7 member 11 (SLC7A11) adversely regulates the Nrf2/HO-1 pathway in ALI, while Nrf2 defensively restrains ferroptosis in ALI caused by seawater submersion [6]. Further studies have reportedly uncovered additional regulatory mechanisms of Nrf2 and ferroptosis in ALI (Fig. 3). Activation of signal transducer and activator of transcription 3 (STAT3) into its phosphorylated form in IIR-ALI led to increased expression of SLC7A11 [63], mitigating the IIR-ALI. Conversely, inhibition of STAT3 expression decreased SLC7A11 expression. Further studies revealed that *Nrf2* knockdown had an activating effect on STAT3, suggesting that Nrf2 and STAT3 co-regulate SLC7A11 to reduce ferroptosis and protect against IIR-ALI. Furthermore, telomerase reverse transcriptase (TERT) with SLC7A11 expression was found to be significantly reduced in lung tissues of *Nrf2*^-/-^ mice with lipid peroxidation accumulation. Conversely, overexpression of TERT led to a reduction in iron accumulation and a notable increase in the levels of ferroptosis-related proteins and SLC7A11. These findings suggest that ferroptosis is regulated by TERT, a crucial factor for telomerase activity [64], within an Nrf2/TERT/SLC7A11 axis, which carries out a protective function against IIR-ALI. Thus, anti-ferroptosis strategies to treat ALI may require stimulation of the Nrf2 signalling pathway. In the same year, it was discovered that the inhibitor of apoptosis-stimulating protein of P53 (IASPP) could be used to treat IIR-ALI. Further investigations demonstrated that IASPP operates as an antioxidant within the cytoplasm, dependent on the translocation of Nrf2 to the nucleus [62]. This mechanism leads to decreased expression of hypoxia-inducible factor (HIF)-1α and Tf while boosting the levels of proteins such as FTH1, NAD(P)H quinone dehydrogenase 1 (NQO1), HO-1, and GPX4. Moreover, this process contributes to the reduction of ferroptosis in IIR-ALI, indicating the potential of IASPP as a drug for the treatment of ALI.

Additionally, in recent studies, deletions of related genes were observed to increase Nrf2 expression and decrease ferroptosis in ALI, providing a protective effect. For example, deletion of the genes encoding BACH1 [65] and JMJD3 [66], negative regulators of Nrf2, upregulated the expression of Nrf2, inhibiting the LPS-induced inflammatory response and ferroptosis, and ameliorating LPS-induced ALI. The mRNA-binding protein Au-rich element RNA-binding factor (AUF1) is essential for inhibiting the inflammatory response and reducing sepsis-related symptoms [67]. Studies have shown that AUF1 overexpression reverses ferroptosis-related indicators and improves the survival rate of sepsis-induced ALI mice. Further studies found that AUF1 interacts with the coding sequence and 3’-untranslated region (UTR) of *Nrf2* to stabilise the gene, whereas AUF1 binding to the 3’-UTR of activating transcription factor 3 (*ATF3*) promotes degradation of the gene, producing an anti-ferroptosis effect and ameliorating ALI [68]. Transmembrane glycoprotein mucin 1 (MUC1) also exhibits Nrf2 regulation, specifically through dimerisation of its transmembrane and cytoplasmic domain (MUC1-C), which represses Keap1 expression while promoting phosphorylation of glycogen synthase kinase 3β (GSK3β). The subsequent translocation of Nrf2 into the nucleus leads to upregulation of GPX4 expression. This indicates that MUC1 mitigates ALI by inhibiting ferroptosis via the GSK3β-Keap1-Nrf2-GPX4 signalling pathway [69]. In summary, the Nrf2 signalling pathway and ferroptosis are crucial in ALI, and elucidation of the specific regulatory mechanisms has important potential value for the future treatment of ALI.

### Drugs that treat ALI by modulating the Nrf2 pathway with ferroptosis

ALI caused by oxidative stress and inflammation [70] has limited therapeutic options, mainly comprising supportive treatments such as mechanical ventilation and nutritional therapy [71, 72]. In addition to natural remedies, which are essential for both preventing and curing the illness, it is crucial that we develop new medications to treat ALI. Obacunone has been shown to reduces ROS content and MDA formation, increases *HO-1* mRNA expression levels, and inhibits ubiquitination of Nrf2 in lung tissues of mice with ALI. The degradation of Nrf2 is also reduced, prolonging its half-life, and decreasing the occurrence of ferroptosis in ALI [73]. As research continues, more and more active ingredients are being used in the treatment of ALI (Table 1). For example, astaxanthin [74], itaconate [75], and irisin [76] all modify the Nrf2 signalling pathway and reduce ferroptosis to exert preventative or curative effects on ALI. It follows that targeted research into drugs that inhibit Nrf2 ubiquitination will lead to the discovery of new therapeutics for the treatment of ALI.

**Table 1:** Therapeutic agents with the potential to improve ALI by suppressing ferroptosis via Nrf2 pathway activation.

Therapeutic drug	Regulatory mechanism	Experimental model	Reference
Obacunone	Inhibiting the Nrf2 ubiquitination pathway increases cytoplasmic Nrf2 content and reduces ferroptosis	ALI model was created 7 h after IP injection of LPS (10 mg/kg) into C57BL/6 mice	[73]
Astaxanthin	Increasing Nrf2 levels in lung tissue reduces inflammation and inhibits ferroptosis	ALI model was induced in BALB/c mice by IP injection of LPS (5 mg/kg), followed by a 6-h interval	[74]
Itaconate	Increased bodily accumulation of Nrf2 upregulates GPX4 protein expression and reduces lipid peroxidation	ALI models in C57BL/6 and *Nrf2*-KO mice were induced by IP injection of LPS (10 mg/kg) for 12 h	[75]
Irisin	Ferroptosis is decreased following overexpression of GPX4 and the rise in Nrf2 and HO-1 mRNA levels	LIRI mouse model was established by 120 min of reperfusion after 60 min of ischaemia in the left lung of C57BL/6 mice	[76]
Ferulic acid	Activation of the Nrf2/HO-1 pathway reduces Fe^2+^ release, thereby protecting against ALI	ALI model was induced in female BALB/c mice using the caecum ligation and puncture method	[116]
Urolithin A	Reducing Keap1 expression activates the Nrf2/HO-1 pathway, which in turn reduces ferroptosis in ALI	C57BL/6 mice were given LPS (10 mg/kg) by tracheal drip infusion for 24 h to create an ALI model	[117]
Quercetin	Quercetin activates the Sirt1/Nrf2/GPX4 pathway to prevent ferroptosis	ALI model was established by giving C57BL/6 mice a 12-h tracheal infusion of LPS (5 mg/kg)	[118]
Fibroblast growth factor 10 (FGF10)	FGF10 reduces ferroptosis by activating the SIRT1-Nrf2 pathway to reduce the synthesis of lipid peroxidation products	Tracheal drip of LPS (5 mg/kg) for 6 h in C57BL/6 mice was used to establish an ALI model	[119]
Tempol	Activation of Nrf2 expression and upregulation of organismal synthesis of GSH reduce ferroptosis in lung epithelial cells	ALI model was produced in BALB/c mice 1 week following an IP injection of LPS (10 mg/kg)	[120]

*IP* intraperitoneal, *LIRI* liver ischaemia–reperfusion injury.

## Role of the Nrf2 pathway and ferroptosis in lung cancer

### Keap1-Nrf2-FSP1-mediated ferroptosis in lung cancer

Studies on non-small cell lung cancer (NSCLC), both preclinical and clinical, have demonstrated tight relationships among the resistance to chemotherapy, radiation therapy, and other agents, and the loss of function of Keap1 and overexpression of Nrf2. Nrf2 overexpression starts out playing a protective role in cancer, but ultimately encourages disease progression and metastasis, while mutations in *Keap1* have been linked to a poor prognosis in individuals with advanced NSCLC receiving treatment [10]. A study using a three-dimensional tumour sphere model combined with CRISPR-Cas9 screening reported that Nrf2 overactivation was essential for lung tumour proliferation and survival, whereas silencing of Nrf2 predisposed spherical cells to ferroptosis, which was also consistent with clinical observations [77]. Additionally, Nrf2 overexpression was shown to activate FSP1, further reducing lipid free radical production by lowering CoQ10 and inhibiting ferroptosis from occurring in lung cancer, mediating resistance to ferroptosis and radiation in *Keap1*-deficient lung cancer cells. Subsequent investigations demonstrated that FSP1, regulated by Nrf2, significantly inhibited lung cancer cell growth and sensitised *Keap1*-deficient cells to ferroptosis by knockdown or inhibition of FSP1 expression in *Keap1* post-mutant lung cancer [46, 78]. It has been shown that a combination of FSP1 inhibitors can effectively treat cancers caused by mutations in the oncogenic Kirsten rat sarcoma viral oncogene homologue (KRAS) gene [79]. Therefore, combining FSP1 inhibitors may represent an effective therapeutic strategy for ferroptosis in *Keap1*-deficient lung cancer and cancers with radioresistance.

### Targeting Nrf2 ubiquitination to mediate ferroptosis in lung cancer

The high expression of Nrf2 is essential for the survival of lung cancer cells, thus strategies to reduce Nrf2 expression may be effective for the treatment of lung cancer, in which ubiquitination plays an important role in the Nrf2 protein degradation pathway. In lung adenocarcinoma (LUAD) cells, the E3 ligase MIB1 was shown to induce ubiquitination of Nrf2 in the Neh2 structural domain, following which the ubiquitinated Nrf2 was degraded via the proteasomal pathway. Furthermore, the reduction of Nrf2 increased the susceptibility of the LUAD cells to ferroptosis [80]. In NSCLC, a deubiquitinating enzyme that stabilises Nrf2 expression by deubiquitinating Nrf2, ubiquitin carboxyl-terminal hydrolase 11 (USP11), was positively correlated with Nrf2 expression [81]. The small molecule RSL3 directly binds to USP11 protein to inactivate it, thereby promoting the ubiquitination and degradation of Nrf2 in lung adenocarcinoma cells, making them more susceptible to ferroptosis. This suggests that, by inhibiting the activity of the deubiquitinating enzyme USP11, the level of oxidative stress in LUAD cells can be increased, promoting cellular ferroptosis [82]. Therefore, targeted suppression of the USP11-Nrf2 axis or promotion of Nrf2 ubiquitination to reduce the high expression of Nrf2 may represent effective strategies to reduce drug resistance and increase the susceptibility to ferroptosis of LUAD cells.

### Nrf2-mediated regulation of metabolic susceptibility to lung cancer and ferroptosis occurrence

A growing body of research suggests that cysteine deprivation increases tumour susceptibility to ferroptosis, but the exact mechanism is unclear. Overexpression of Nrf2 in NSCLC activates downstream antioxidant factors that protect lung cancer cells from ferroptosis. However, a negative regulatory interaction between Nrf2 and focadhesin (FOCAD), an adhesion plaque protein that enhances the sensitivity of NSCLC cells to ferroptosis under cysteine deprivation conditions, has been discovered [83]. Further studies revealed that replication protein A1 (RPA1) competes with sMAF for binding to Nrf2, activates the cancer-related FOCAD gene, and upregulates focal adhesion kinase (FAK) activity to make NSCLC cells more susceptible to cysteine deprivation-induced ferroptosis, without affecting GPX4 inhibition-induced ferroptosis. Cysteine deprivation-induced ferroptosis has a significant impact on NSCLC cells, particularly in terms of sensitivity. Alternatively, studies have shown that ferroptosis induced by cysteine deprivation in NSCLC cells with high-level expression of Nrf2 exerts a protective effect independent of GSH [84]. High Nrf2 expression increases the catalytic subunit activity of glutamate-cysteine ligase (GCLC). Nrf2 is a critical transcriptional regulator of GCLC. In NSCLC deprived of cysteine, GCLC replaces cysteine with other small uncharged amino acids to produce *γ*-glutamyl-peptide. Thus, inhibiting glutamate buildup prevents iron prolapse. Additionally, lung cancer cells defective in Keap1 are glucose dependent; when glucose levels are low, high uptake of cystine by lung cancer cells via the Nrf2/SLC7A11 axis stimulates the accumulation of intracellular disulfide bonds and depletion of NADPH. This leads to ferroptosis, but can be reversed by inhibition of Nrf2 [85]. Elucidating the regulatory mechanisms of Nrf2 sensitivity to ferroptosis in lung cancer cells under cysteine or glucose limitation may lead to novel therapeutic strategies in the clinical management of NSCLC.

### Drugs that mediate ferroptosis in lung cancer via the Nrf2 pathway

Lung cancer has the highest incidence and mortality rates of all cancer types globally and is still on the rise [86]. China is predicted to have substantially higher rates of incidence and mortality from lung cancer than any other country between 2015 and 2030 [87], and the mortality rate is predicted to rise by ~40%, necessitating the urgent development of new lung cancer treatments, such as those listed in Table 2 [88]. Studies have shown that nuclear Nrf2 is overexpressed in 26% of NSCLC tumours [89]. Lung cancer cells become much more susceptible to ferroptosis under Nrf2 silencing. For instance, manoalide and trabectedin have been shown to induce Fe²⁺ overload by modulating the Nrf2/SLC7A11 axis, thereby triggering ferroptosis in lung cancer cells [90, 91]. Additionally, a novel ferroptosis inducer, S-3′-hydroxy-7′, 2′, 4′-trimethoxyisoxane (ShtIX), selectively eliminates NSCLC cells while sparing normal cells. Further research indicated that ShtIX induces ferroptosis in NSCLC cells by blocking the Nrf2/HO-1 signalling pathway [92].

**Table 2:** Therapeutic agents with the potential to induce ferroptosis in lung cancer cells via inhibition of Nrf2.

Therapeutic drug	Regulatory mechanism	Experimental model	Reference(s)
RSL3	RSL3 suppresses USP11 expression, which allows Nrf2 protein to be degraded, leading to ferroptosis in KLK lung cancer cells	SC injection of A549 cells into nude mice established xenograft lung cancer models with tumours measuring 100 mm^3^	[82]
Ginkgetin	Ginkgetin downregulates the Nrf2/HO-1 pathway, lowering the expression of SLC7A11 and GPX4, and encouraging cellular ferroptosis	Establishment of a nude mouse model of NSCLC transplantation by SC injection of A549 cell tumours (~100 mm^3^)	[88]
Manoalide	Ferroptosis is produced via stimulation of the mitochondrial Ca^2+^ overload-driven FTH1 pathway and inhibition of the KRAS-ERK pathway and the Nrf2-SLC7A11 axis	Tumour tissue was taken at 12 weeks for organoid culture after adenovirus infection via tracheal drip	[90]
Trabectedin	Elevations in Fe^2+^ and ROS trigger apoptosis by stimulating the HIF-1/IRP1 axis and transferrin receptor protein 1, and blocking the suppression of the Keap1/Nrf2 axis	A549, H460, PC-9, H1299, and HSAECs cells were selected for mechanistic studies	[91]
ShtIX	Inhibition of the Nrf2/GPX4 pathway in lung cancer cells induces cellular ferroptosis and increases lipid peroxide and Fe^2+^ levels	Xenograft lung cancer model was established by SC injection of A549 cells into nude mice, resulting in tumour xenografts of ~ 100 mm^3^ in size	[92]
Erastin/sorafenib	Erastin/sorafenib induces ferroptosis in cisplatin-resistant NSCLC cells through inhibition of the Nrf2/xCT pathway	SC injection of N5CP cells into nude mice and 600 mm^3^ xenografted tumours were used to establish a xenograft cisplatin-resistant tumour model	[94]
Cisplatin and PRLX93936	Inhibition of Nrf2 expression promotes ferroptosis in NSCLC cells, synergizing with cisplatin and PRLX93936 to enhance GPX4 inhibition	Cell lines A549 and H23 were selected for mechanistic studies in NSCLC cells	[95]
Acetaminophen	Erastin synergistically inhibits the Nrf2/HO-1 signalling pathway with acetaminophen and promotes lipid peroxidation in A549 cells	SC injection of A549 cells into the right side of the thymus of BALB/c nude mice generated tumours ≤ 80 mm^3^, establishing a xenograft lung cancer model	[96]
ZVI-NP	ZVI-NP promotes ferroptosis in lung cancer cells by activating the AMPK/mTOR pathway, which enhances degradation of Nrf2 via the GSK3/β-TrCP pathway	SC implantation of A549 cells in NOD/SCID mice to establish an A549 xenograft model and a spontaneous lung metastasis model in immunodeficient mice	[121]
Isoorientin	Cellular ferroptosis is aided by inhibition of the SIRT6/Nrf2/GPX4 signalling pathway	Six days after SC injection of A549/DDP tumour cells into BALB/c-nu mice, average tumour diameter reached 0.5 cm	[122]
Metformin and eriocitrin	Iron overload in lung cancer cells is facilitated by increased MDA, ROS, and iron ion levels, coupled with reduced expression of GPX4, SLC7A11, Nrf2, and HO-1 proteins	A549 and H1299 cells were selected for mechanistic studies	[123, 124]
Cephaeline	Targeted reduction of Nrf2 expression in lung cancer cells causes lipid peroxidation	Injection of H460 cells into the right dorsal side of BALB/c-nu mice was used to establish a SC tumour model	[114]
Clobetasol propionate	Inhibition of Nrf2 expression makes human lung cancer cells more radiosensitive, increasing mitochondrial ROS and lipid peroxidation	Xenograft tumour model was created by giving NOD/SCID BALB/c female mice SC injections in the right hind leg, producing tumours of ≤ 100 mm^3^ in size	[125]

*AMPK* AMP-activated protein kinase, *β-TrCP* beta-transducin repeat containing protein, *ERK* extracellular signal-regulated kinase, *IRP1* iron regulatory protein 1, *mTOR* mammalian target of rapamycin, *NOD/SCID* non-obese diabetic/severe combined immunodeficiency mutant, *SC* subcutaneous, *ZVI-NP* zero-valent-iron nanoparticle.

Additionally, *Keap1* mutations are commonly found in NSCLCs such as LUAD and lung squamous carcinoma, and are strongly linked to poor prognosis and resistance to current treatments [55, 56, 93]. In a study using microRNA (miR)-6077-targeted inhibition of *Keap1* expression, the resulting initiation of antioxidant genes by Nrf2 was found to be a key factor in the inhibition of ferroptosis and generating cisplatin resistance in LUAD. Indeed, a combination of drugs targeting the sponging of miR-6077 effectively increased the sensitivity of LUAD cells to cisplatin [93]. Thus, the use of combined medications to address the drug resistance arising from *Keap1* mutations represents a promising novel approach to combat chemotherapy resistance in clinical settings.

Studies have shown that activation of the Nrf2/HO-1 pathway contributes to cisplatin resistance. Ginkgetin disrupts cellular redox balance in cisplatin-treated cells by increasing ROS levels and suppressing the Nrf2/HO-1 pathway. This results in reduced expression of SLC7A11 and GPX4 [88]. Furthermore, co-administration of ginkgetin and cisplatin enhance cytotoxicity in NSCLC cells, consistent with recent findings indicating that cisplatin-resistant NSCLC cells might also be eliminated effectively with low-dose cisplatin combined with the ferroptosis agonist erastin/sorafenib [94]. Additionally, the erastin analogue PRLX93936 in combination with cisplatin was found to induce ferroptosis of NSCLC cells in a clinical trial. Further studies found that cisplatin and PRLX93936 co-treatment inhibited GPX4 overexpression, which was potentiated by *Nrf2* knockdown [95]. Increasing the sensitivity of cancer cells to erastin may likewise address the problem of erastin-induced resistance to ferroptosis. Both acetaminophen and MT1DP induce ferroptosis in cancer cells by reducing Nrf2 expression, thereby enhancing lipid peroxidation and sensitizing NSCLC cells to erastin [96, 97]. Clearly, Nrf2 plays a significant role in both anti-lung cancer treatment and resistance, indicating that Nrf2 inhibitors combined with other medications could be part of a successful treatment plan for lung cancer.

## NRF2 pathway and the role of ferroptosis in other lung diseases

### Particulate matter-mediated lung injury triggered by the Nrf2 and ferroptosis pathways

One of the most common types of ambient particulate matter is PM_2.5_, which is a type of fine particulate matter with a diameter less than or equal to 2.5 μm that poses a significant threat to human health. Extensive research indicates that PM_2.5_ can infiltrate the human respiratory system, disrupt pulmonary gas exchange, and contribute to the development and progression of respiratory disorders. Recent studies exploring ferroptosis have further revealed that exposure to PM_2.5_ induces oxidative stress in mouse lungs. This oxidative stress is characterised by elevated levels of GSH and MDA, along with reduced expression of antioxidant enzymes such as SOD2, GPX4, and SLC7A11. Additionally, exposure to PM_2.5_ upregulates the expression of fibrosis-related proteins (e.g. α-SMA) and collagen [98]. PM_2.5_ can induce ferroptosis in lung cells, causing lung lesions that are mainly associated with inhibition of the Nrf2 signalling pathway. Thus, therapeutic approaches aimed at controlling the Nrf2 signalling pathway could mitigate PM_2.5_-induced lung injury by alleviating ferroptosis in pulmonary tissues. Astragaloside IV has been shown to be beneficial in attenuating PM_2.5_-induced lung injury. Astragaloside IV exhibits pharmacological effects that include anti-inflammatory and antioxidant properties, and enhances the expression of ferroptosis-related proteins GPX4 and SLC7A11, thereby regulating cellular iron transport and mitigating ferroptosis [99]. Furthermore, Astragaloside IV reduces the levels of pro-inflammatory cytokines interleukin (IL)-6, IL-1β, and tumour necrosis factor-α by modulating the Nrf2 signalling pathway. Melatonin and tectoridin reportedly have the same beneficial effects [100, 101]. Sipeimine and rosavin prevent ferroptosis by reducing the levels of Fe^2+^, MDA, and inflammatory factors, offering therapeutic benefits in lung injury through phosphoinositide-3-kinase (PI3K)/protein kinase B (AKT)-mediated Nrf2 expression. Research indicates that AKT enhances Nrf2 expression and regulates ferroptosis in lung injury [102]. However, this therapeutic effect is reversed by administration of the PI3K inhibitor LY294002, suggesting that sipeimine combined with rosavin regulates ferroptosis via the PI3K/AKT/Nrf2 pathway, addressing PM_2.5_-induced damage to the lungs [103]. Given the link between PM_2.5_-induced oxidative stress and the onset of ferroptosis in the lungs, enhancing the Nrf2 signalling pathway appears to be crucially important for preventing ferroptosis. Consequently, medications that regulate Nrf2/ferroptosis may 1 day be used to treat lung disorders brought on by PM_2.5_.

### Radiation-induced lung injury and pulmonary fibrosis

The most frequent and dangerous side effect of chest radiation therapy is lung damage, leading to conditions such as radiation-induced pulmonary fibrosis [104, 105]. The main mechanism for this condition is a radiation-triggered series of inflammatory responses that ultimately result in cellular demise [106]. Increasing research has revealed that GPX4 expression is dramatically decreased in lung tissues after radiation irradiation, suggesting the occurrence of ferroptosis. However, following administration of the ferroptosis inhibitors liproxstatin-1 or ferrostatin-1, the levels of inflammatory factors and ROS are reduced, endogenous antioxidant pathways related to Nrf2, HO-1, and NQO1 are upregulated, transforming growth factor-β expression is downregulated, and iron ptosis is reduced in lung tissue [107]. Further studies revealed that p62 could facilitate the nuclear translocation of Nrf2 by interacting with Keap1, a promotional effect that could be cancelled by siRNA against *Keap1* [108]. Therefore, the reduction of ferroptosis through the p62-Keap1-Nrf2 pathway could serve as a useful treatment for radiological lung injury or fibrosis.

### Other lung diseases

**Fig. 4:**
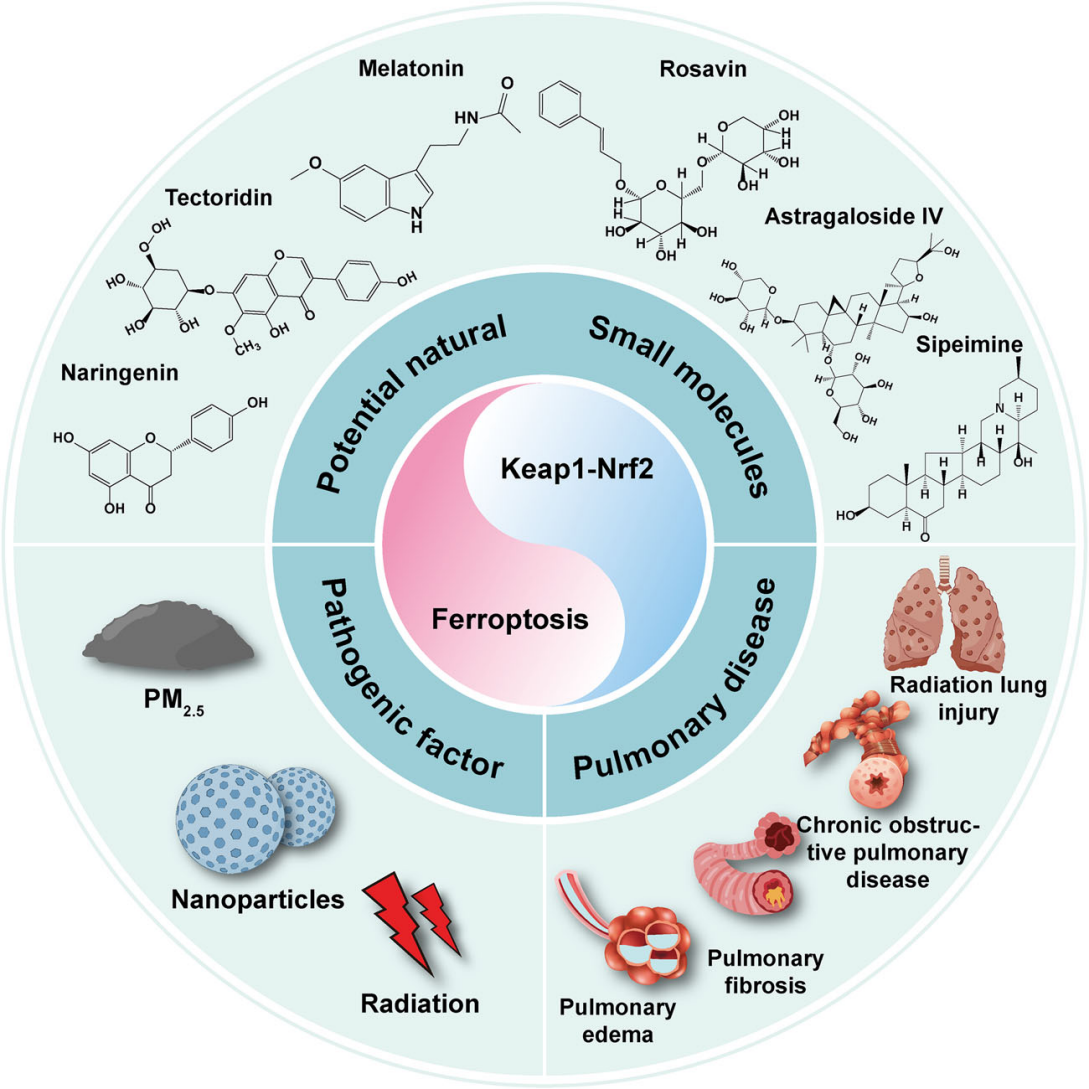
The interaction of Keap1-Nrf2 and ferroptosis with various lung diseases, and potential therapeutic agents.

Nrf2 is implicated in the pathogenesis and progression of various pulmonary diseases, including pulmonary fibrosis (PF), high-altitude pulmonary oedema [107], plateau pulmonary oedema, and chronic obstructive pulmonary disease (COPD) [109]. According to one study [110], lung tissues from COPD patients exhibit ferroptosis due to hypermethylation of the CpG region of the *Nrf2* promoter, which in turn prevents expression of Nrf2/GPX4. Additionally, ferroptosis was found to occur in either plateau-type pulmonary oedema or paraquat-induced PF, whereas regulation of the Keap1/Nrf2/HO-1 pathway significantly upregulated ferroptosis-associated proteins and reversed ferroptosis [109, 111]. An in-depth study found that Nrf2-mediated regulation of ferroptosis may be related to mitochondrial ROS. By blocking mitochondrial ROS production, treatment with Mito-TEMPO successfully lowers RSL3 toxicity, increases cell antioxidant capacity, restores GPX4 expression, and prevents ferroptosis [112]. Although these findings indicate that the regulation of ferroptosis by Nrf2 in lung diseases may be related to the inhibition of mitochondrial ROS, further validation studies are necessary. We anticipate that elucidating the intrinsic link between the Nrf2 pathway and ferroptosis will be the key to treating lung diseases, as illustrated in Fig. 4.

## Summary and discussion

**Fig. 5:**
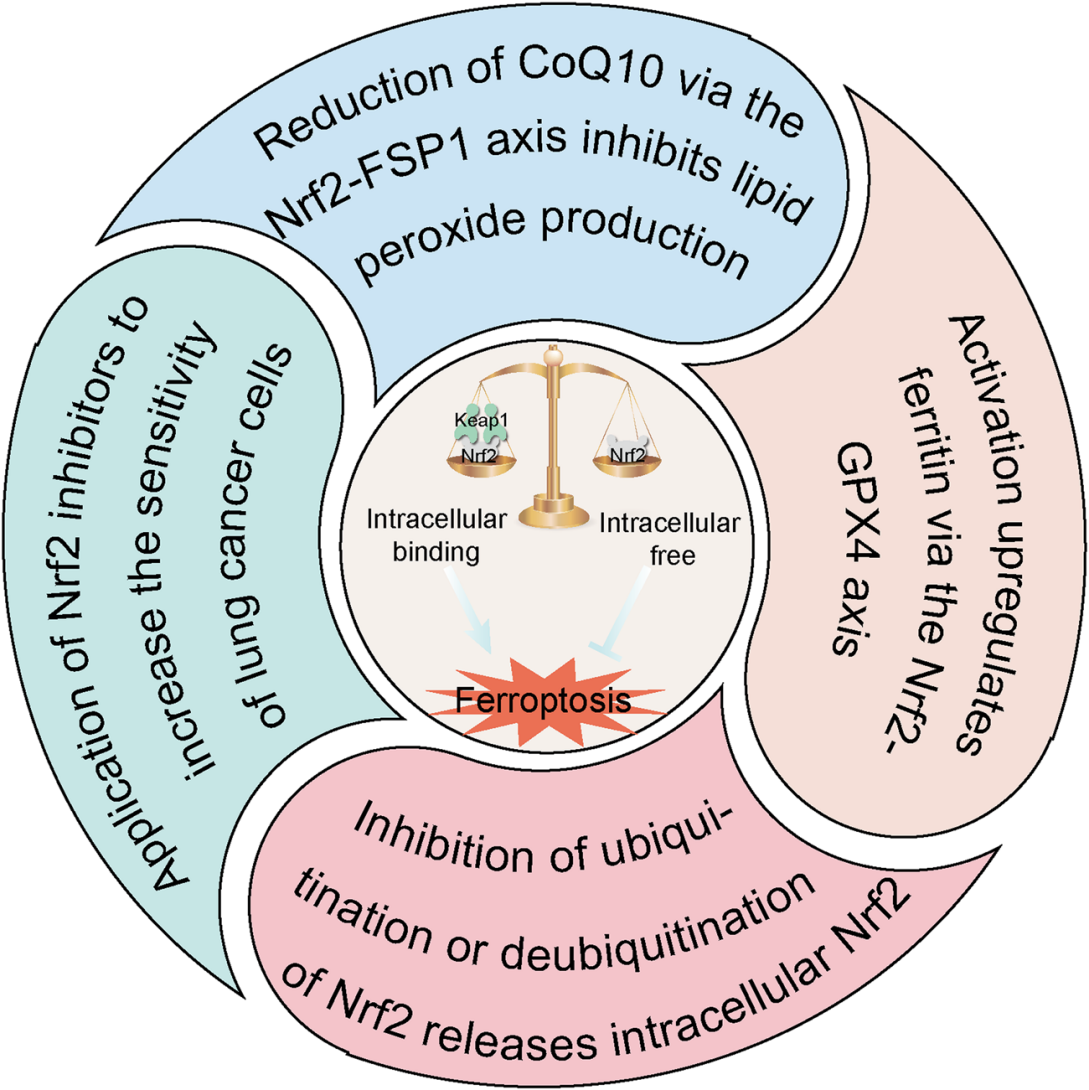
Mechanisms of drugs that mediate Nrf2 to regulate ferroptosis in the treatment of lung diseases.

Nrf2 plays an important role in the regulation of ferroptosis, which exhibits a dual role in lung diseases by modulating ferroptosis [113]. To prevent oxidative stress and the inflammatory response in acute and chronic progressive diseases, approaches to enhance the expression of Nrf2 and thereby inhibit ferroptosis should undoubtedly play a central role in treating ALI, COPD, and idiopathic PF in the future. By contrast, as therapeutic strategies for lung cancer have gradually developed, it has become clear how detrimental Nrf2 overexpression is to the treatment of lung cancer. Furthermore, Nrf2 overexpression raises the expression levels of FSP1, GPX4, SLC7A11, and other related proteins in lung cancer [114, 115], and obstructs the therapeutic process of ferroptosis activators by shielding lung cancer cells from ferroptosis. Therefore, in the treatment of lung tumours with *Keap1* deletion or Nrf2 overexpression, Nrf2 inhibitors should be administered to enhance the susceptibility of lung cancer cells to ferroptosis. Based on currently available research, there are three main mechanisms by which medications interact with Nrf2 to impact ferroptosis in lung disease (Fig. 5): (1) reduction of CoQ10 through the Nrf2-FSP1 axis to inhibit the generation of lipid peroxides, thereby mitigating the occurrence of cellular ferroptosis; (2) enhancement of antioxidant proteins (e.g. HO-1 and NQO1) via the crucially important Nrf2-GPX4 axis alongside increased protein expression of GPX4 and FTH to counteract ferroptosis; and (3) increased Nrf2 levels in lung tissues to prevent ubiquitination or deubiquitination of Nrf2, thereby facilitating its nuclear translocation and inhibiting ferroptosis. We predict that selective activation or inhibition of Nrf2 (as appropriate for different lung diseases), supplemented by drug therapy, will likely improve drug efficacy while reducing cellular tolerance to ferroptosis, and will become a major treatment approach for lung diseases in the future.

## References

[1] Wang Q, Unwalla H, Rahman I. Dysregulation of mitochondrial complexes and dynamics by chronic cigarette smoke exposure utilizing MitoQC reporter mice. Mitochondrion. 2022;63:43–50. 35032706 10.1016/j.mito.2022.01.003 PMC8885972

[2] Gould GS, Hurst JR, Trofor A, Alison JA, Fox G, Kulkarni MM, et al. Recognising the importance of chronic lung disease: a consensus statement from the global alliance for chronic diseases (Lung Diseases group). Respir Res. 2023;24:15. 36639661 10.1186/s12931-022-02297-y PMC9838069

[3] Claxton S, Porter P, Brisbane J, Bear N, Wood J, Peltonen V, et al. Identifying acute exacerbations of chronic obstructive pulmonary disease using patient-reported symptoms and cough feature analysis. NPJ Digit Med. 2021;4:107. 34215828 10.1038/s41746-021-00472-x PMC8253790

[4] Lahiri A, Maji A, Potdar PD, Singh N, Parikh P, Bisht B, et al. Lung cancer immunotherapy: progress, pitfalls, and promises. Mol Cancer. 2023;22:40. 36810079 10.1186/s12943-023-01740-y PMC9942077

[5] Werder RB, Liu T, Abo KM, Lindstrom-Vautrin J, Villacorta-Martin C, Huang J, et al. CRISPR interference interrogation of COPD GWAS genes reveals the functional significance of desmoplakin in iPSC-derived alveolar epithelial cells. Sci Adv. 2022;8:eabo6566. 35857525 10.1126/sciadv.abo6566 PMC9278866

[6] Qiu YB, Wan BB, Liu G, Wu YX, Chen D, Lu MD, et al. Nrf2 protects against seawater drowning-induced acute lung injury via inhibiting ferroptosis. Respir Res. 2020;21:232. 32907551 10.1186/s12931-020-01500-2 PMC7488337

[7] Xu S, Zhou Y, Yu L, Huang X, Huang J, Wang K, et al. Protective effect of eurotium cristatum fermented loose dark tea and eurotium cristatum particle on MAPK and PXR/AhR signaling pathways induced by electronic cigarette exposure in mice. Nutrients. 2022;14:2843. 35889800 10.3390/nu14142843 PMC9318283

[8] Kato K, Papageorgiou I, Shin YJ, Kleinhenz JM, Palumbo S, Hahn S, et al. Lung-targeted delivery of dimethyl fumarate promotes the reversal of age-dependent established lung fibrosis. Antioxid (Basel). 2022;11:492. 10.3390/antiox11030492 PMC8944574 35326142

[9] Pei Z, Qin Y, Fu X, Yang F, Huo F, Liang X, et al. Inhibition of ferroptosis and iron accumulation alleviates pulmonary fibrosis in a bleomycin model. Redox Biol. 2022;57:102509. 36302319 10.1016/j.redox.2022.102509 PMC9614651

[10] Scalera S, Mazzotta M, Cortile C, Krasniqi E, De Maria R, Cappuzzo F, et al. KEAP1-mutant NSCLC: the catastrophic failure of a cell-protecting hub. J Thorac Oncol. 2022;17:751–7. 35351670 10.1016/j.jtho.2022.03.011

[11] Moi P, Chan K, Asunis I, Cao A, Kan YW. Isolation of NF-E2-related factor 2 (Nrf2), a NF-E2-like basic leucine zipper transcriptional activator that binds to the tandem NF-E2/AP1 repeat of the beta-globin locus control region. Proc Natl Acad Sci USA. 1994;91:9926–30. 7937919 10.1073/pnas.91.21.9926 PMC44930

[12] Zhang D, Ren Y, He Y, Chang R, Guo S, Ma S, et al. In situ forming and biocompatible hyaluronic acid hydrogel with reactive oxygen species-scavenging activity to improve traumatic brain injury repair by suppressing oxidative stress and neuroinflammation. Mater Today Bio. 2022;15:100278. 35601897 10.1016/j.mtbio.2022.100278 PMC9119840

[13] Ngo V, Karunatilleke NC, Brickenden A, Choy WY, Duennwald ML. Oxidative stress-induced misfolding and inclusion formation of Nrf2 and Keap1. Antioxid (Basel). 2022;11:243. 10.3390/antiox11020243 PMC8868093 35204126

[14] Pillai R, Hayashi M, Zavitsanou AM, Papagiannakopoulos T. NRF2: KEAPing tumors protected. Cancer Discov. 2022;12:625–43. 35101864 10.1158/2159-8290.CD-21-0922 PMC8904278

[15] Herrera-Bravo J, Beltrán JF, Huard N, Saavedra K, Saavedra N, Alvear M, et al. Anthocyanins found in pinot noir waste induce target genes related to the Nrf2 signalling in endothelial cells. Antioxid (Basel). 2022;11:1239. 10.3390/antiox11071239 PMC9311808 35883728

[16] Ngo V, Brickenden A, Liu H, Yeung C, Choy WY, Duennwald ML. A novel yeast model detects Nrf2 and Keap1 interactions with Hsp90. Dis Model Mech. 2022;15:dmm049235. 35088844 10.1242/dmm.049258 PMC9016900

[17] Kopacz A, Klóska D, Proniewski B, Cysewski D, Personnic N, Piechota-Polańczyk A, et al. Keap1 controls protein S-nitrosation and apoptosis-senescence switch in endothelial cells. Redox Biol. 2020;28:101304. 31491600 10.1016/j.redox.2019.101304 PMC6731384

[18] Yao H, Zhang W, Yang F, Ai F, Du D, Li Y. Discovery of caffeoylisocitric acid as a Keap1-dependent Nrf2 activator and its effects in mesangial cells under high glucose. J Enzym Inhib Med Chem. 2022;37:178–88. 10.1080/14756366.2021.1998025 PMC8667952 34894983

[19] Shao N, Huang H, Idris M, Peng X, Xu F, Dong S, et al. KEAP1 mutations drive tumorigenesis by suppressing SOX9 ubiquitination and degradation. Adv Sci (Weinh). 2020;7:2001018. 33173725 10.1002/advs.202001018 PMC7610265

[20] Liu S, Pi J, Zhang Q. Mathematical modeling reveals quantitative properties of KEAP1-NRF2 signaling. Redox Biol. 2021;47:102139. 34600335 10.1016/j.redox.2021.102139 PMC8531862

[21] Sánchez-de-Diego C, Pedrazza L, Pimenta-Lopes C, Martinez-Martinez A, Dahdah N, Valer JA, et al. NRF2 function in osteocytes is required for bone homeostasis and drives osteocytic gene expression. Redox Biol. 2021;40:101845. 33373776 10.1016/j.redox.2020.101845 PMC7773566

[22] Zhang X, Ye L, Xu H, Zhou Q, Tan B, Yi Q, et al. NRF2 is required for structural and metabolic maturation of human induced pluripotent stem cell-derived ardiomyocytes. Stem Cell Res Ther. 2021;12:208. 33762018 10.1186/s13287-021-02264-2 PMC7992990

[23] Uruno A, Matsumaru D, Ryoke R, Saito R, Kadoguchi S, Saigusa D, et al. Nrf2 suppresses oxidative stress and inflammation in app lnock-in alzheimer’s disease model mice. Mol Cell Biol. 2020;40:e00467–00419. 31932477 10.1128/MCB.00467-19 PMC7048263

[24] Lee DH, Park JS, Lee YS, Han J, Lee DK, Kwon SW, et al. SQSTM1/p62 activates NFE2L2/NRF2 via ULK1-mediated autophagic KEAP1 degradation and protects mouse liver from lipotoxicity. Autophagy. 2020;16:1949–73. 31913745 10.1080/15548627.2020.1712108 PMC7595589

[25] Yamamoto M, Kensler TW, Motohashi H. The KEAP1-NRF2 system: a thiol-based sensor-effector apparatus for maintaining redox homeostasis. Physiol Rev. 2018;98:1169–203. 29717933 10.1152/physrev.00023.2017 PMC9762786

[26] Wang X, Ma B, Wen X, You H, Sheng C, Bu L, et al. Correction: Bone morphogenetic protein 4 alleviates nonalcoholic steatohepatitis by inhibiting hepatic ferroptosis. Cell Death Discov. 2022;8:463. 36424399 10.1038/s41420-022-01246-4 PMC9691719

[27] Li P, Jiang M, Li K, Li H, Zhou Y, Xiao X, et al. Glutathione peroxidase 4-regulated neutrophil ferroptosis induces systemic autoimmunity. Nat Immunol. 2021;22:1107–17. 34385713 10.1038/s41590-021-00993-3 PMC8609402

[28] Huang YB, Jiang L, Liu XQ, Wang X, Gao L, Zeng HX, et al. Melatonin alleviates acute kidney injury by inhibiting NRF2/Slc7a11 axis-mediated ferroptosis. Oxid Med Cell Longev. 2022;2022:4776243. 35979396 10.1155/2022/4776243 PMC9377938

[29] Zhang Z, Lu M, Chen C, Tong X, Li Y, Yang K, et al. Holo-lactoferrin: the link between ferroptosis and radiotherapy in triple-negative breast cancer. Theranostics. 2021;11:3167–82. 33537080 10.7150/thno.52028 PMC7847686

[30] Meng X, Lu Z, Zhang L, Wang Z. A pH/ATP-responsive nanomedicine via disrupting multipath homeostasis of ferroptosis for enhanced cancer therapy. Chem Eng J. 2023;457:141313.

[31] Ouyang S, Li H, Lou L, Huang Q, Zhang Z, Mo J, et al. Inhibition of STAT3-ferroptosis negative regulatory axis suppresses tumor growth and alleviates chemoresistance in gastric cancer. Redox Biol. 2022;52:102317. 35483272 10.1016/j.redox.2022.102317 PMC9108091

[32] Luo Y, Huang S, Wei J, Zhou H, Wang W, Yang J, et al. Long noncoding RNA LINC01606 protects colon cancer cells from ferroptotic cell death and promotes stemness by SCD1-Wnt/β-catenin-TFE3 feedback loop signalling. Clin Transl Med. 2022;12:e752. 35485210 10.1002/ctm2.752 PMC9052012

[33] Balihodzic A, Prinz F, Dengler MA, Calin GA, Jost PJ, Pichler M. Non-coding RNAs and ferroptosis: potential implications for cancer therapy. Cell Death Differ. 2022;29:1094–106. 35422492 10.1038/s41418-022-00998-x PMC9177660

[34] Beatty A, Singh T, Tyurina YY, Tyurin VA, Samovich S, Nicolas E, et al. Ferroptotic cell death triggered by conjugated linolenic acids is mediated by ACSL1. Nat Commun. 2021;12:2244. 33854057 10.1038/s41467-021-22471-y PMC8046803

[35] Xie L, Chen W, Chen Q, Jiang Y, Song E, Zhu X, et al. Synergistic hydroxyl radical formation, system XC- inhibition and heat shock protein crosslinking tango in ferrotherapy: a prove-of-concept study of “sword and shield” theory. Mater Today Bio. 2022;16:100353. 35865409 10.1016/j.mtbio.2022.100353 PMC9294558

[36] Xie Y, Kang R, Klionsky DJ, Tang D. GPX4 in cell death, autophagy, and disease. Autophagy. 2023;19:2621–38. 37272058 10.1080/15548627.2023.2218764 PMC10472888

[37] Mao C, Liu X, Zhang Y, Lei G, Yan Y, Lee H, et al. DHODH-mediated ferroptosis defence is a targetable vulnerability in cancer. Nature. 2021;593:586–90. 33981038 10.1038/s41586-021-03539-7 PMC8895686

[38] Lv Y, Liang C, Sun Q, Zhu J, Xu H, Li X, et al. Structural insights into FSP1 catalysis and ferroptosis inhibition. Nat Commun. 2023;14:5933. 37739943 10.1038/s41467-023-41626-7 PMC10516921

[39] Arslanbaeva L, Tosi G, Ravazzolo M, Simonato M, Tucci FA, Pece S, et al. UBIAD1 and CoQ10 protect melanoma cells from lipid peroxidation-mediated cell death. Redox Biol. 2022;51:102272. 35255427 10.1016/j.redox.2022.102272 PMC8902599

[40] Bersuker K, Hendricks JM, Li Z, Magtanong L, Ford B, Tang PH, et al. The CoQ oxidoreductase FSP1 acts parallel to GPX4 to inhibit ferroptosis. Nature. 2019;575:688–92. 31634900 10.1038/s41586-019-1705-2 PMC6883167

[41] Dixon SJ, Olzmann JA. The cell biology of ferroptosis. Nat Rev Mol Cell Biol. 2024;25:424–42. 38366038 10.1038/s41580-024-00703-5 PMC12187608

[42] Feng W, Xiao Y, Zhao C, Zhang Z, Liu W, Ma J, et al. New deferric amine compounds efficiently chelate excess iron to treat Iron overload disorders and to prevent ferroptosis. Adv Sci (Weinh). 2022;9:e2202679. 36031399 10.1002/advs.202202679 PMC9561787

[43] Xiu Z, Zhu Y, Han J, Li Y, Yang X, Yang G, et al. Caryophyllene oxide induces ferritinophagy by regulating the NCOA4/FTH1/LC3 pathway in hepatocellular carcinoma. Front Pharm. 2022;13:930958. 10.3389/fphar.2022.930958 PMC9313605 35899120

[44] Cheng Y, Gao Y, Li J, Rui T, Li Q, Chen H, et al. TrkB agonist N-acetyl serotonin promotes functional recovery after traumatic brain injury by suppressing ferroptosis via the PI3K/Akt/Nrf2/Ferritin H pathway. Free Radic Biol Med. 2023;194:184–98. 36493983 10.1016/j.freeradbiomed.2022.12.002

[45] Liu W, Wang L, Liu C, Dai Z, Li T, Tang B. Edaravone ameliorates cerebral ischemia-reperfusion injury by downregulating ferroptosis via the Nrf2/FPN pathway in rats. Biol Pharm Bull. 2022;45:1269–75. 36047195 10.1248/bpb.b22-00186

[46] Koppula P, Lei G, Zhang Y, Yan Y, Mao C, Kondiparthi L, et al. A targetable CoQ-FSP1 axis drives ferroptosis- and radiation-resistance in KEAP1 inactive lung cancers. Nat Commun. 2022;13:2206. 35459868 10.1038/s41467-022-29905-1 PMC9033817

[47] Song JX, An JR, Chen Q, Yang XY, Jia CL, Xu S, et al. Liraglutide attenuates hepatic iron levels and ferroptosis in db/db mice. Bioengineered. 2022;13:8334–48. 35311455 10.1080/21655979.2022.2051858 PMC9161873

[48] Hong JY, Kim H, Lee J, Jeon WJ, Lee YJ, Ha IH. Harpagophytum procumbens inhibits iron overload-onduced oxidative stress through activation of Nrf2 signaling in a rat model of lumbar spinal stenosis. Oxid Med Cell Longev. 2022;2022:3472443. 36160714 10.1155/2022/3472443 PMC9492433

[49] Feng Z, Min L, Chen H, Deng W, Tan M, Liu H, et al. Iron overload in the motor cortex induces neuronal ferroptosis following spinal cord injury. Redox Biol. 2021;43:101984. 33933882 10.1016/j.redox.2021.101984 PMC8105676

[50] Seiwert N, Wecklein S, Demuth P, Hasselwander S, Kemper TA, Schwerdtle T, et al. Heme oxygenase 1 protects human colonocytes against ROS formation, oxidative DNA damage and cytotoxicity induced by heme iron, but not inorganic iron. Cell Death Dis. 2020;11:787. 32968051 10.1038/s41419-020-02950-8 PMC7511955

[51] Yang J, Mo J, Dai J, Ye C, Cen W, Zheng X, et al. Cetuximab promotes RSL3-induced ferroptosis by suppressing the Nrf2/HO-1 signalling pathway in KRAS mutant colorectal cancer. Cell Death Dis. 2021;12:1079. 34775496 10.1038/s41419-021-04367-3 PMC8590697

[52] Chen Y, Guo X, Zeng Y, Mo X, Hong S, He H, et al. Oxidative stress induces mitochondrial iron overload and ferroptotic cell death. Sci Rep. 2023;13:15515. 37726294 10.1038/s41598-023-42760-4 PMC10509277

[53] Foggetti G, Li C, Cai H, Hellyer JA, Lin WY, Ayeni D, et al. Genetic determinants of EGFR-driven lung cancer growth and therapeutic response in vivo. Cancer Discov. 2021;11:1736–53. 33707235 10.1158/2159-8290.CD-20-1385 PMC8530463

[54] Liu Y, Tao S, Liao L, Li Y, Li H, Li Z, et al. TRIM25 promotes the cell survival and growth of hepatocellular carcinoma through targeting Keap1-Nrf2 pathway. Nat Commun. 2020;11:348. 31953436 10.1038/s41467-019-14190-2 PMC6969153

[55] Jeong Y, Hoang NT, Lovejoy A, Stehr H, Newman AM, Gentles AJ, et al. Role of KEAP1/NRF2 and TP53 mutations in lung squamous cell carcinoma development and radiation resistance. Cancer Discov. 2017;7:86–101. 27663899 10.1158/2159-8290.CD-16-0127 PMC5222718

[56] Collisson EA, Campbell JD, Brooks AN, Berger AH, Lee W, Chmielecki J, et al. Comprehensive molecular profiling of lung adenocarcinoma. Nature. 2014;511:543–50. 25079552 10.1038/nature13385 PMC4231481

[57] Lee YT, Chen YL, Wu YH, Chen IS, Chang HS, Wang YH, et al. Meso-dihydroguaiaretic acid ameliorates acute respiratory distress syndrome through inhibiting neutrophilic inflammation and scavenging free radical. Antioxid (Basel). 2022;11:123. 10.3390/antiox11010123 PMC8772954 35052627

[58] Chen Z, Dong WH, Chen Q, Li QG, Qiu ZM. Downregulation of miR-199a-3p mediated by the CtBP2-HDAC1-FOXP3 transcriptional complex contributes to acute lung injury by targeting NLRP1. Int J Biol Sci. 2019;15:2627–40. 31754335 10.7150/ijbs.37133 PMC6854378

[59] Kochanek M, Kochanek J, Böll B, Eichenauer DA, Beutel G, Bracht H, et al. Veno-venous extracorporeal membrane oxygenation (vv-ECMO) for severe respiratory failure in adult cancer patients: a retrospective multicenter analysis. Intensive Care Med. 2022;48:332–42. 35146534 10.1007/s00134-022-06635-y PMC8866383

[60] Kan X, Chen Y, Huang B, Fu S, Guo W, Ran X, et al. Effect of Palrnatine on lipopolysaccharide-induced acute lung injury by inhibiting activation of the Akt/NF‍-κB pathway. J Zhejiang Univ Sci B. 2021;22:929–40. 34783223 10.1631/jzus.B2000583 PMC8593526

[61] Breau M, Houssaini A, Lipskaia L, Abid S, Born E, Marcos E, et al. The antioxidant N-acetylcysteine protects from lung emphysema but induces lung adenocarcinoma in mice. JCI Insight. 2019;4:e127647. 31578304 10.1172/jci.insight.127647 PMC6795405

[62] Li Y, Cao Y, Xiao J, Shang J, Tan Q, Ping F, et al. Inhibitor of apoptosis-stimulating protein of p53 inhibits ferroptosis and alleviates intestinal ischemia/reperfusion-induced acute lung injury. Cell Death Differ. 2020;27:2635–50. 32203170 10.1038/s41418-020-0528-x PMC7429834

[63] Qiang Z, Dong H, Xia Y, Chai D, Hu R, Jiang H. Nrf2 and STAT3 alleviates ferroptosis-mediated IIR-ALI by regulating SLC7A11. Oxid Med Cell Longev. 2020;2020:5146982. 33014271 10.1155/2020/5146982 PMC7520693

[64] Dong H, Xia Y, Jin S, Xue C, Wang Y, Hu R, et al. Nrf2 attenuates ferroptosis-mediated IIR-ALI by modulating TERT and SLC7A11. Cell Death Dis. 2021;12:1027. 34716298 10.1038/s41419-021-04307-1 PMC8556385

[65] Wang RX, Gu X, Zhang SX, Zhao YJ, Zhang HJ, Li FY. Deletion of BACH1 alleviates ferroptosis and protects against LPS-triggered acute lung injury by activating Nrf2/HO-1 signaling pathway. Biochem Biophys Res Commun. 2023;644:8–14. 36621150 10.1016/j.bbrc.2023.01.002

[66] Peng J, Fan B, Bao C, Jing C. JMJD3 deficiency alleviates lipopolysaccharide‑induced acute lung injury by inhibiting alveolar epithelial ferroptosis in a Nrf2‑dependent manner. Mol Med Rep. 2021;24:807. 34542160 10.3892/mmr.2021.12447

[67] Lu JY, Sadri N, Schneider RJ. Endotoxic shock in AUF1 knockout mice mediated by failure to degrade proinflammatory cytokine mRNAs. Genes Dev. 2006;20:3174–84. 17085481 10.1101/gad.1467606 PMC1635151

[68] Wang Y, Chen D, Xie H, Jia M, Sun X, Peng F, et al. AUF1 protects against ferroptosis to alleviate sepsis-induced acute lung injury by regulating NRF2 and ATF3. Cell Mol Life Sci. 2022;79:228. 35391558 10.1007/s00018-022-04248-8 PMC11072094

[69] Wang YM, Gong FC, Qi X, Zheng YJ, Zheng XT, Chen Y, et al. Mucin 1 inhibits ferroptosis and sensitizes vitamin E to alleviate sepsis-induced acute lung injury through GSK3β/Keap1-Nrf2-GPX4 pathway. Oxid Med Cell Longev. 2022;2022:2405943. 35910848 10.1155/2022/2405943 PMC9334047

[70] Yeh CH, Yang JJ, Yang ML, Li YC, Kuan YH. Rutin decreases lipopolysaccharide-induced acute lung injury via inhibition of oxidative stress and the MAPK-NF-κB pathway. Free Radic Biol Med. 2014;69:249–57. 24486341 10.1016/j.freeradbiomed.2014.01.028

[71] Chen Y, Zhang Y, Li N, Jiang Z, Li X. Role of mitochondrial stress and the NLRP3 inflammasome in lung diseases. Inflamm Res. 2023;72:829–46. 36905430 10.1007/s00011-023-01712-4 PMC10007669

[72] Li J, Lu K, Sun F, Tan S, Zhang X, Sheng W, et al. Panaxydol attenuates ferroptosis against LPS-induced acute lung injury in mice by Keap1-Nrf2/HO-1 pathway. J Transl Med. 2021;19:96. 33653364 10.1186/s12967-021-02745-1 PMC7927246

[73] Li J, Deng SH, Li J, Li L, Zhang F, Zou Y, et al. Obacunone alleviates ferroptosis during lipopolysaccharide-induced acute lung injury by upregulating Nrf2-dependent antioxidant responses. Cell Mol Biol Lett. 2022;27:29. 35305560 10.1186/s11658-022-00318-8 PMC8933916

[74] Luo L, Huang F, Zhong S, Ding R, Su J, Li X. Astaxanthin attenuates ferroptosis via Keap1-Nrf2/HO-1 signaling pathways in LPS-induced acute lung injury. Life Sci. 2022;311:121091. 36252699 10.1016/j.lfs.2022.121091

[75] He R, Liu B, Xiong R, Geng B, Meng H, Lin W, et al. Itaconate inhibits ferroptosis of macrophage via Nrf2 pathways against sepsis-induced acute lung injury. Cell Death Discov. 2022;8:43. 35110526 10.1038/s41420-021-00807-3 PMC8810876

[76] Wang Y, Dong Z, Zhang Z, Wang Y, Yang K, Li X. Postconditioning with irisin attenuates lung ischemia/reperfusion injury by suppressing ferroptosis via induction of the Nrf2/HO-1 signal axis. Oxid Med Cell Longev. 2022;2022:9911167. 35281462 10.1155/2022/9911167 PMC8906956

[77] Takahashi N, Cho P, Selfors LM, Kuiken HJ, Kaul R, Fujiwara T, et al. 3D culture models with CRISPR screens reveal hyperactive NRF2 as a prerequisite for spheroid formation via regulation of proliferation and ferroptosis. Mol Cell. 2020;80:828–844.e826. 33128871 10.1016/j.molcel.2020.10.010 PMC7718371

[78] Emmanuel N, Li H, Chen J, Zhang Y. FSP1, a novel KEAP1/NRF2 target gene regulating ferroptosis and radioresistance in lung cancers. Oncotarget. 2022;13:1136–9. 36264074 10.18632/oncotarget.28301 PMC9584440

[79] Müller F, Lim JKM, Bebber CM, Seidel E, Tishina S, Dahlhaus A, et al. Elevated FSP1 protects KRAS-mutated cells from ferroptosis during tumor initiation. Cell Death Differ. 2023;30:442–56. 36443441 10.1038/s41418-022-01096-8 PMC9950476

[80] Wang H, Huang Q, Xia J, Cheng S, Pei D, Zhang X, et al. The E3 ligase MIB1 promotes proteasomal degradation of NRF2 and sensitizes lung cancer cells to ferroptosis. Mol Cancer Res. 2022;20:253–64. 34670864 10.1158/1541-7786.MCR-21-0342

[81] Meng C, Zhan J, Chen D, Shao G, Zhang H, Gu W, et al. The deubiquitinase USP11 regulates cell proliferation and ferroptotic cell death via stabilization of NRF2 USP11 deubiquitinates and stabilizes NRF2. Oncogene. 2021;40:1706–20. 33531626 10.1038/s41388-021-01660-5

[82] Zhang W, Li X, Xu J, Wang Y, Xing Z, Hu S, et al. The RSL3 induction of KLK lung adenocarcinoma cell ferroptosis by inhibition of USP11 activity and the NRF2-GSH axis. Cancers (Basel). 2022;14:5233. 36358651 10.3390/cancers14215233 PMC9658295

[83] Liu P, Wu D, Duan J, Xiao H, Zhou Y, Zhao L, et al. NRF2 regulates the sensitivity of human NSCLC cells to cystine deprivation-induced ferroptosis via FOCAD-FAK signaling pathway. Redox Biol. 2020;37:101702. 32898818 10.1016/j.redox.2020.101702 PMC7486457

[84] Kang YP, Mockabee-Macias A, Jiang C, Falzone A, Prieto-Farigua N, Stone E, et al. Non-canonical glutamate-cysteine ligase activity protects against ferroptosis. Cell Metab. 2021;33:174–189.e177. 33357455 10.1016/j.cmet.2020.12.007 PMC7839835

[85] Koppula P, Olszewski K, Zhang Y, Kondiparthi L, Liu X, Lei G, et al. KEAP1 deficiency drives glucose dependency and sensitizes lung cancer cells and tumors to GLUT inhibition. iScience. 2021;24:102649. 34151236 10.1016/j.isci.2021.102649 PMC8193145

[86] Zhang N, Wu Y, Wu Y, Wang L, Chen J, Wang X, et al. Ferroptosis-related genes are potential therapeutic targets and the model of these genes influences overall survival of NSCLC patients. Cells. 2022;11:2207. 35883650 10.3390/cells11142207 PMC9319237

[87] Wang N, Gao Q, Tang J, Jiang Y, Yang L, Shi X, et al. Anti-tumor effect of local injectable hydrogel-loaded endostatin alone and in combination with radiotherapy for lung cancer. Drug Deliv. 2021;28:183–94. 33427520 10.1080/10717544.2020.1869864 PMC7808389

[88] Lou JS, Zhao LP, Huang ZH, Chen XY, Xu JT, Tai WC, et al. Ginkgetin derived from Ginkgo biloba leaves enhances the therapeutic effect of cisplatin via ferroptosis-mediated disruption of the Nrf2/HO-1 axis in EGFR wild-type non-small-cell lung cancer. Phytomedicine. 2021;80:153370. 33113504 10.1016/j.phymed.2020.153370

[89] Roh JL, Kim EH, Jang H, Shin D. Nrf2 inhibition reverses the resistance of cisplatin-resistant head and neck cancer cells to artesunate-induced ferroptosis. Redox Biol. 2017;11:254–62. 28012440 10.1016/j.redox.2016.12.010 PMC5198738

[90] Ni Y, Liu J, Zeng L, Yang Y, Liu L, Yao M, et al. Natural product manoalide promotes EGFR-TKI sensitivity of lung cancer cells by KRAS-ERK pathway and mitochondrial Ca^2+^ overload-induced ferroptosis. Front Pharm. 2022;13:1109822. 10.3389/fphar.2022.1109822 PMC9873971 36712673

[91] Cai S, Ding Z, Liu X, Zeng J. Trabectedin induces ferroptosis via regulation of HIF-1α/IRP1/TFR1 and Keap1/Nrf2/GPX4 axis in non-small cell lung cancer cells. Chem Biol Interact. 2023;369:110262. 36396105 10.1016/j.cbi.2022.110262

[92] Chen J, Zhou S, Zhang X, Zhao H. S-3’-hydroxy-7’, 2’, 4’-trimethoxyisoxane, a novel ferroptosis inducer, promotes NSCLC cell death through inhibiting Nrf2/HO-1 signaling pathway. Front Pharm. 2022;13:973611. 10.3389/fphar.2022.973611 PMC9465255 36105203

[93] Bi G, Liang J, Zhao M, Zhang H, Jin X, Lu T, et al. MiR-6077 promotes cisplatin/pemetrexed resistance in lung adenocarcinoma via CDKN1A/cell cycle arrest and KEAP1/ferroptosis pathways. Mol Ther Nucleic Acids. 2022;28:366–86. 35505963 10.1016/j.omtn.2022.03.020 PMC9035384

[94] Li Y, Yan H, Xu X, Liu H, Wu C, Zhao L. Erastin/sorafenib induces cisplatin-resistant non-small cell lung cancer cell ferroptosis through inhibition of the Nrf2/xCT pathway. Oncol Lett. 2020;19:323–33. 31897145 10.3892/ol.2019.11066 PMC6923844

[95] Liang Z, Zhao W, Li X, Wang L, Meng L, Yu R. Cisplatin synergizes with PRLX93936 to induce ferroptosis in non-small cell lung cancer cells. Biochem Biophys Res Commun. 2021;569:79–85. 34237431 10.1016/j.bbrc.2021.06.088

[96] Gai C, Yu M, Li Z, Wang Y, Ding D, Zheng J, et al. Acetaminophen sensitizing erastin-induced ferroptosis via modulation of Nrf2/heme oxygenase-1 signaling pathway in non-small-cell lung cancer. J Cell Physiol. 2020;235:3329–39. 31541463 10.1002/jcp.29221 PMC13482668

[97] Gai C, Liu C, Wu X, Yu M, Zheng J, Zhang W, et al. MT1DP loaded by folate-modified liposomes sensitizes erastin-induced ferroptosis via regulating miR-365a-3p/NRF2 axis in non-small cell lung cancer cells. Cell Death Dis. 2020;11:751. 32929075 10.1038/s41419-020-02939-3 PMC7490417

[98] Li J, Mao M, Li J, Chen Z, Ji Y, Kong J, et al. Oral administration of omega-3 fatty acids attenuates lung injury caused by PM_2.5_ respiratory inhalation simply and feasibly in vivo. Int J Mol Sci. 2022;23:5323. 35628131 10.3390/ijms23105323 PMC9140442

[99] Wang X, Wang Y, Huang D, Shi S, Pei C, Wu Y, et al. Astragaloside IV regulates the ferroptosis signaling pathway via the Nrf2/SLC7A11/GPX4 axis to inhibit PM_2.5_-mediated lung injury in mice. Int Immunopharmacol. 2022;112:109186. 36115280 10.1016/j.intimp.2022.109186

[100] Guohua F, Tieyuan Z, Xinping M, Juan X. Melatonin protects against PM_2.5_-induced lung injury by inhibiting ferroptosis of lung epithelial cells in a Nrf2-dependent manner. Ecotoxicol Environ Saf. 2021;223:112588. 34364124 10.1016/j.ecoenv.2021.112588

[101] Dong T, Fan X, Zheng N, Yan K, Hou T, Peng L, et al. Activation of Nrf2 signalling pathway by tectoridin protects against ferroptosis in particulate matter-induced lung injury. Br J Pharm. 2023;180:2532–49. 10.1111/bph.16085 37005797

[102] Wang Y, Shen Z, Zhao S, Huang D, Wang X, Wu Y, et al. Sipeimine ameliorates PM_2.5_-induced lung injury by inhibiting ferroptosis via the PI3K/Akt/Nrf2 pathway: a network pharmacology approach. Ecotoxicol Environ Saf. 2022;239:113615. 35567927 10.1016/j.ecoenv.2022.113615

[103] Wang Y, Zhao S, Jia N, Shen Z, Huang D, Wang X, et al. Pretreatment with rosavin attenuates PM_2.5_-induced lung injury in rats through antiferroptosis via PI3K/Akt/Nrf2 signaling pathway. Phytother Res. 2023;37:195–210. 36097321 10.1002/ptr.7606

[104] Marks LB, Yu X, Vujaskovic Z, Small W, Folz R, Anscher MS. Radiation-induced lung injury. Semin Radiat Oncol. 2003;13:333–45. 12903021 10.1016/S1053-4296(03)00034-1

[105] Ghafoori P, Marks LB, Vujaskovic Z, Kelsey CR. Radiation-induced lung injury. Assess Manag Prev Oncol (Williston Park) 2008;22:37–47. 18251282

[106] Giuranno L, Roig EM, Wansleeben C, van den Berg A, Groot AJ, Dubois L, et al. NOTCH inhibition promotes bronchial stem cell renewal and epithelial barrier integrity after irradiation. Stem Cells Transl Med. 2020;9:799–812. 32297712 10.1002/sctm.19-0278 PMC7308641

[107] Li X, Duan L, Yuan S, Zhuang X, Qiao T, He J. Ferroptosis inhibitor alleviates radiation-induced lung fibrosis (RILF) via down-regulation of TGF-β1. J Inflamm (Lond). 2019;16:11. 31160885 10.1186/s12950-019-0216-0 PMC6542066

[108] Li X, Chen J, Yuan S, Zhuang X, Qiao T. Activation of the P62-Keap1-NRF2 pathway protects against ferroptosis in radiation-induced lung injury. Oxid Med Cell Longev. 2022;2022:8973509. 35847598 10.1155/2022/8973509 PMC9277166

[109] Song CY, Feng MX, Li L, Wang P, Lu X, Lu YQ. Tripterygium wilfordii Hook.f. ameliorates paraquat-induced lung injury by reducing oxidative stress and ferroptosis via Nrf2/HO-1 pathway. Ecotoxicol Environ Saf. 2023;252:114575. 36706526 10.1016/j.ecoenv.2023.114575

[110] Zhang Z, Fu C, Liu J, Sai X, Qin C, Di T, et al. Hypermethylation of the Nrf2 promoter induces ferroptosis by inhibiting the Nrf2-GPX4 axis in COPD. Int J Chron Obstruct Pulmon Dis. 2021;16:3347–62. 34934311 10.2147/COPD.S340113 PMC8684379

[111] Wang Y, Shen Z, Pei C, Zhao S, Jia N, Huang D, et al. Eleutheroside B ameliorated high altitude pulmonary edema by attenuating ferroptosis and necroptosis through Nrf2-antioxidant response signaling. Biomed Pharmacother. 2022;156:113982. 36411652 10.1016/j.biopha.2022.113982

[112] Taufani IP, Situmorang JH, Febriansah R, Tasminatun S, Sunarno S, Yang LY, et al. Mitochondrial ROS induced by ML385, an Nrf2 inhibitor aggravates the ferroptosis induced by RSL3 in human lung epithelial BEAS-2B cells. Hum Exp Toxicol. 2023;42:9603271221149663. 36625148 10.1177/09603271221149663

[113] Zhang L, Xu Y, Cheng Z, Zhao J, Wang M, Sun Y, et al. The EGR1/miR-139/NRF2 axis orchestrates radiosensitivity of non-small-cell lung cancer via ferroptosis. Cancer Lett. 2024;595:217000. 38821254 10.1016/j.canlet.2024.217000

[114] Chen P, Ye Q, Liang S, Zeng L. Cephaeline promotes ferroptosis by targeting NRF2 to exert anti-lung cancer efficacy. Pharm Biol. 2024;62:195–206. 38339810 10.1080/13880209.2024.2309891 PMC10860416

[115] Chen Y, Jiang Z, Zhang C, Zhang L, Chen H, Xiao N, et al. 5-Methylcytosine transferase NSUN2 drives NRF2-mediated ferroptosis resistance in non-small cell lung cancer. J Biol Chem. 2024;300:106793. 38403250 10.1016/j.jbc.2024.106793 PMC11065752

[116] Tang X, Liu J, Yao S, Zheng J, Gong X, Xiao B. Ferulic acid alleviates alveolar epithelial barrier dysfunction in sepsis-induced acute lung injury by activating the Nrf2/HO-1 pathway and inhibiting ferroptosis. Pharm Biol. 2022;60:2286–94. 36433644 10.1080/13880209.2022.2147549 PMC9707381

[117] Lou L, Wang M, He J, Yang S, Meng F, Wang S, et al. Urolithin A (UA) attenuates ferroptosis in LPS-induced acute lung injury in mice by upregulating Keap1-Nrf2/HO-1 signaling pathway. Front Pharm. 2023;14:1067402. 10.3389/fphar.2023.1067402 PMC10034769 36969874

[118] Deng S, Li J, Li L, Lin S, Yang Y, Liu T, et al. Quercetin alleviates lipopolysaccharide‑induced acute lung injury by inhibiting ferroptosis via the Sirt1/Nrf2/Gpx4 pathway. Int J Mol Med. 2023;52:118. 37888753 10.3892/ijmm.2023.5321 PMC10635686

[119] Lin L, Yang L, Wang N, Chen S, Du X, Chen R, et al. FGF10 protects against LPS-induced epithelial barrier injury and inflammation by inhibiting SIRT1-ferroptosis pathway in acute lung injury in mice. Int Immunopharmacol. 2024;127:111426. 38147776 10.1016/j.intimp.2023.111426

[120] Yu Y, Liang J, Yuan Z, Wang A, Liu X, Chen Y, et al. Bioactive compound schaftoside from Clinacanthus nutans attenuates acute liver injury by inhibiting ferroptosis through activation the Nrf2/GPX4 pathway. J Ethnopharmacol. 2024;328:118135. 38556139 10.1016/j.jep.2024.118135

[121] Hsieh CH, Hsieh HC, Shih FS, Wang PW, Yang LX, Shieh DB, et al. An innovative NRF2 nano-modulator induces lung cancer ferroptosis and elicits an immunostimulatory tumor microenvironment. Theranostics. 2021;11:7072–91. 34093872 10.7150/thno.57803 PMC8171079

[122] Feng S, Li Y, Huang H, Huang H, Duan Y, Yuan Z, et al. Isoorientin reverses lung cancer drug resistance by promoting ferroptosis via the SIRT6/Nrf2/GPX4 signaling pathway. Eur J Pharm. 2023;954:175853. 10.1016/j.ejphar.2023.175853 37329975

[123] Deng C, Xiong L, Chen Y, Wu K, Wu J. Metformin induces ferroptosis through the Nrf2/HO-1 signaling in lung cancer. BMC Pulm Med. 2023;23:360. 37749553 10.1186/s12890-023-02655-6 PMC10521546

[124] Gao M, Lai K, Deng Y, Lu Z, Song C, Wang W, et al. Eriocitrin inhibits epithelial-mesenchymal transformation (EMT) in lung adenocarcinoma cells via triggering ferroptosis. Aging. 2023;15:10089–104. 37787987 10.18632/aging.205049 PMC10599723

[125] Rai A, Patwardhan RS, Jayakumar S, Pachpatil P, Das D, Panigrahi GC, et al. Clobetasol propionate, a Nrf-2 inhibitor, sensitizes human lung cancer cells to radiation-induced killing via mitochondrial ROS-dependent ferroptosis. Acta Pharm Sin. 2024;45:1506–19. 10.1038/s41401-024-01233-8 PMC11192725 38480835

